# Variability in Ultrasound Backscatter Induced by Trabecular Microstructure Deterioration in Cancellous Bone

**DOI:** 10.1155/2018/4786329

**Published:** 2018-01-29

**Authors:** Xingxing Chou, Feng Xu, Ying Li, Chengcheng Liu, Dean Ta, Lawrence H. Le

**Affiliations:** ^1^Department of Electronic Engineering, Fudan University, Shanghai 200433, China; ^2^Institute of Acoustics, Tongji University, No. 1239 Siping Road, Shanghai 200092, China; ^3^State Key Laboratory of ASIC and System, Fudan University, Shanghai 200433, China; ^4^Key Laboratory of Medical Imaging Computing and Computer Assisted Intervention (MICCAI) of Shanghai, Shanghai 200032, China; ^5^Department of Radiology and Diagnostic Imaging, University of Alberta, Edmonton, AB, Canada

## Abstract

To determine the relationship between the ultrasonic backscatter parameters and trabecular microstructural variations in cancellous bone, three erosion procedures were performed to simulate various changes in the cancellous bone microstructure. The finite difference time domain (FDTD) method was used to simulate the backscatter signal in cancellous bone. Ultrasonic backscatter properties were derived as functions of the porosity when the ultrasound incident directions were perpendicular and parallel to the major trabeculae direction (MTD), respectively. The variability in the apparent backscatter coefficient (ABC) and apparent integrated backscatter (AIB) due to the trabecular microstructure was revealed. Significant negative correlations between the backscatter parameters (ABC and AIB) and the porosity of the cancellous bone were observed. The simulations showed that the ABC and AIB were influenced by the direction of the trabecular microstructural variations. The linear regressions between the ultrasonic backscatter parameters (ABC and AIB) and the porosity showed significantly different slopes for three erosion procedures when they are ultrasonically perpendicular (for ABC, −1.22 dB, −0.98 dB, and −0.46 dB; for AIB, −0.74 dB, −0.69 dB, and −0.25 dB) and parallel (for ABC, −1.87 dB, −0.69 dB, and −0.51 dB; for AIB, −0.9 dB, −0.5 dB, and −0.34 dB) to the MTD. This paper investigated the relationship between ultrasonic backscatter and cancellous bone microstructure deterioration and indicated that the ultrasonic backscatter could be affected by cancellous bone microstructure deterioration direction.

## 1. Introduction

Osteoporosis is a multifactorial skeletal disease characterized by decreased bone mass and deteriorated microarchitecture that leads to an increased risk of fracture [1]. Early detection and treatment of osteoporosis are essential for decreasing the risk of fracture. Lots of situations can lead to osteoporosis, such as age-related and microgravity-related situations [2–6]. Both aggravating trend of aging population and the aerospace development indicate the importance of early detection and treatment of osteoporosis.

Ultrasonic backscatter has shown great advantages and potential as a noninvasive tool for cancellous bone assessment [7–15]. Compared with ultrasonic through-transmission measurement, the backscatter measurement can be performed in pulse-echo mode with a single transducer and has easier access to skeletal sites such as the hip and spine. Hosokawa has studied the changes of the ultrasonic through-transmission signal [16]. In theory, the backscatter signal could provide more microstructural information; the backscatter signal is closely related to cancellous bone properties, including the bone mineral density (BMD), bone volume fraction (BV/TV), trabecular separation (Tb.Sp), ultimate strength, and Young's modulus [7–14, 17–25].

Bone is a tissue undergoing continuous construction and degradation; the location of cancellous bone in people's body and different bone loss and growth processes determine the various cancellous bone microstructure. The trabecular orientation of bone tissue changes in response to mechanical stimuli; the process of bone loss destruction and reconstruction is anisotropic [26, 27]. In age-related osteoporosis, the trabecular elements perpendicular to the major trabecular direction (MTD) are more strongly lost than those parallel to the MTD. Because the weakly oriented trabecular elements to which large loads are not usually applied are the first to disappear, and the porosity of the bone increases (and the state of the osteoporosis progresses) [28, 29]. For spaceflight-induced bone loss, both the weak and strong oriented trabecular elements to which loads are not applied disappear rapidly [1, 4, 5, 28].

The ultrasonic backscattering and propagation are substantially affected by the cancellous bone microstructure [19]; thus the reliability needs to be further improved in ultrasonic backscatter apparatuses, especially assessment of the bone mass changes during bone loss and growth, because the relationship of the ultrasonic backscatter and cancellous bone microstructure parameters is not yet clearly understood. However, a detailed investigation on the relationship between the ultrasonic backscatter and cancellous bone microstructure is difficult because of the various cancellous bone microstructure in the bone loss process. Some image erosion algorithms have been used to simulate the degradation of cancellous bones [16, 29, 30]. Hosokawa realized various cancellous bone microstructure using image erosion methods [16, 29]. Three erosion procedures correspond to age-related, microgravity-related, and other reasons related to bone loss [16, 29]. This paper cited their algorithms to simulate the degradation of cancellous bone in normal bone loss, weightlessness, or microgravity environment bone loss. The ultrasonic backscatter parameters, such as apparent backscatter coefficient (ABC) and apparent integrated backscatter (AIB), are generally measured from fixed region of interest in the ultrasonic backscatter signal [21]. A detailed investigation on the relationship between the ultrasonic backscatter and cancellous bone microstructure is needed.

The objective of this study is to investigate the variability in ultrasonic backscatter induced by different deteriorations of trabecular microstructure. Image erosion methods were used to simulate the deteriorations of trabecular microstructure, and three erosion procedures were performed to realize deteriorations in the cancellous bone microstructure. The FDTD method was used to simulate the backscatter signal in cancellous bone. The variability in the ABC and AIB due to the deteriorations of trabecular microstructure was revealed.

## 2. Methods

The reconstruction of the 3D microcomputed tomographic (*μ*-CT) images is useful for the numerical analysis of cancellous bone [32, 33]. The finite difference time domain (FDTD) method is useful for simulating the ultrasound propagation in cancellous bone [29, 34]. The cancellous bone model for the FDTD simulation was realized by the reconstruction of the 3D microcomputed tomographic (*μ*-CT) images from the cancellous bone [35].

### 2.1. Cancellous Bone Erosion or Deteriorations

A cancellous bone specimen (approximately 20 × 20 × 10 mm) was sawed from a bovine distal tibia, and the trabecular image was provided by a *μ*-CT system (skyscan1076, Bruker micro-CT, Belgium) with a spatial resolution of 36.4 *μ*m.

**Figure 1:**
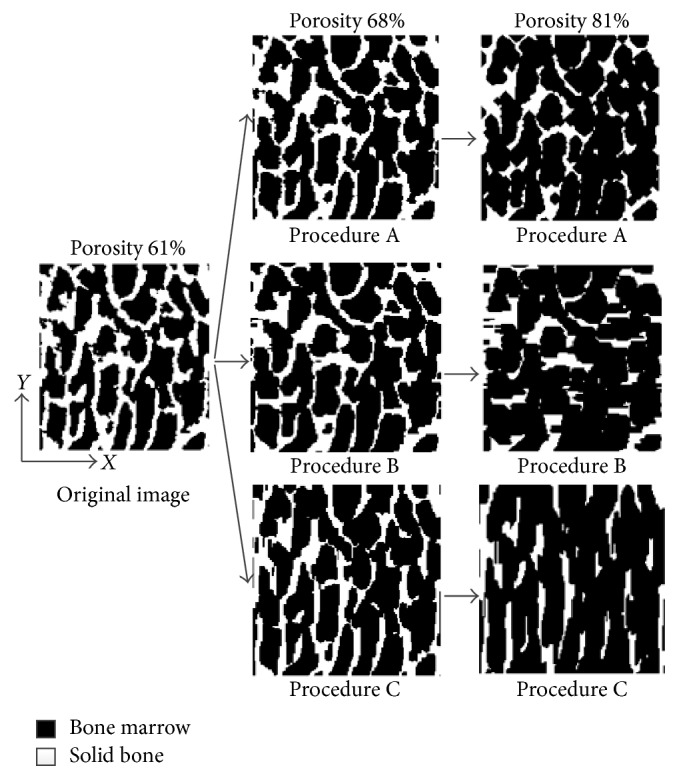
Deteriorations in trabecular microstructure simulated by three erosion procedures.

The binary image was obtained from the gray image by the automatic threshold function in the MATLAB to clearly distinguish between the trabeculae and bone marrow. The trabecular structure is with a MTD in most normal cancellous bone [36]. The 2D trabecular structures of the cancellous bone model are defined in *x*-*y* plane. As shown in Figure 1, a MTD along the *y*-direction can be observed.

An image erosion technique was used to erode the edges of the trabeculae in the cancellous bone model and from which to simulate the bone loss process [30, 35]. The erosion procedure was to transform the solid bone into bone marrow [16]. The porosity increased by an increment approximately 2% at the same time. The trabeculae was eroded by three erosion procedures named A, B, and C; each of the erosion procedures was applied in different direction of the trabecular edges, from which to realize distinct changing processes of the trabecular microstructure in different bone loss processes. In erosion procedure A, the erosion was randomly distributed in every direction [16]. In the other two procedures B and C, the erosions were distributed in the *y*- and *x*-direction, respectively. Procedure C realized the age-related bone loss, procedure B realized the spaceflight-induced bone loss, and procedure A is for any other reason.

**Figure 2:**
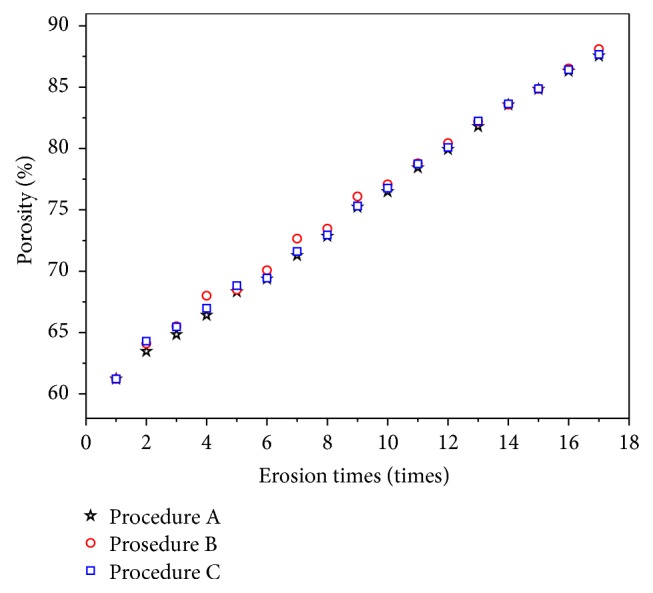
Porosity of cancellous bone with respect to erosion times.

The direction of the erosion distributed was set in the erosion function. To the three erosion procedures, as an example, Figure 1 shows the different changes in trabecular structure induced by them. In Figure 1, the image in the left with a 61% porosity is original image before erosion, and the porosity is increasing with the erosion of original image. As an example, when the porosity is 68%, the images of three procedures are shown in the middle and their porosities are the same; compared with the original image, in the image of procedure A (top), the erosion is randomly distributed in every direction. In the other two procedures B (middle) and C (below), the erosions were distributed in the *y*- and *x*-direction, respectively. The solid bones (white) decrease in the corresponding direction. When the porosity is 81% (right), the difference of three erosion procedures is more obvious. It appears that the trabeculae changes of the three erosion procedures are different in directions. Thus, various trabecular microstructures of different causes could be realized by the three erosion procedures, and the trabecular orientation (or the pore orientation) in the *y*-direction becomes stronger in the cancellous bone model. The porosities of all the cancellous bone models eroded in the three procedures are shown in Figure 2. Each erosion procedure is performed 16 times; porosity of cancellous bone before erosion is 61%, increased by an increment approximately 2% at the same time and increased from 60% to 90% based on the general range.

### 2.2. Ultrasonic Backscatter Simulations

**Figure 3:**
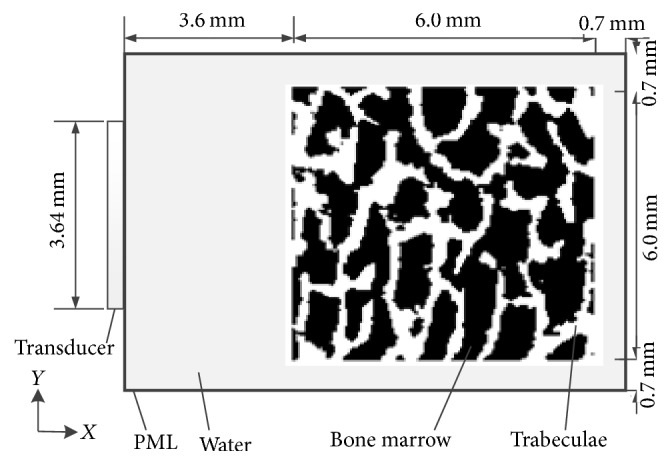
The geometry of the simulation model.

Figure 3 shows the FDTD simulation model, with a total region of 10.5 × 7.4 mm, for the ultrasonic backscatter measurement, and a cancellous bone model (6.0 × 6.0 mm) was placed in the center [21]. The transmitting surfaces were with a diameter of 3.64 mm. The physical parameters of the simulation model are listed in Table 1.

As shown in Figure 3, the ultrasonic propagation was along the *x*-direction. With the cancellous bone model rotated by 90 degrees, the ultrasonic propagation is perpendicular to the MTD of the cancellous bone when the MTD is in *y*-direction.

A Gaussian-modulated sinusoidal pulse was used as the ultrasound pressure source [21]:

(1) $$\begin{array}{c} p\left(\;t\;\right)=-t\cdot \exp\left(\;-4\beta^{2}t^{2}\;\right)\sin\left(\;2\pi f_{0}t\;\right), \end{array}$$

where *β* is the bandwidth and *f*_0_ is central frequency. In the simulation, the parameters are defined as follows: *β* = 0.5 MHz; the central frequency *f*_0_ was set to 1 MHz; the space step was set to 36.4 *μ*m, corresponding to the voxel size of the cancellous bone image; and the time step was 5 ns [37].

### 2.3. Backscatter Signal Analysis

**Figure 4:**
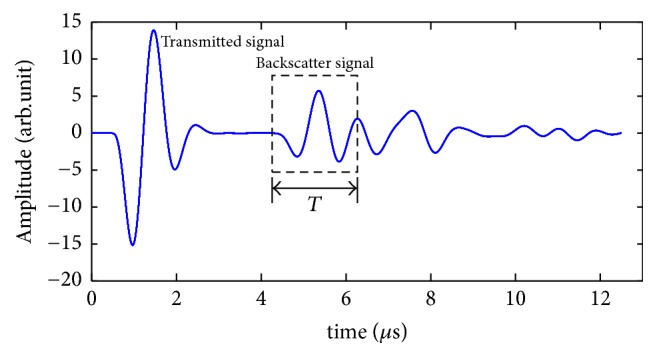
Simulated backscatter signals and signal of interest selection.

Figure 4 shows a typical simulated backscatter signal at 1 MHz. The backscattered signal of interest (SOI) was selected by a rectangular window of *T* in length of the backscatter signal, where *T* = 2 *μ*s [20].

ABC and AIB were defined as follows [7, 11, 38, 39]:

(2) ABC=8.68ln⁡SSOIfSrf,AIB=1fmax−fmin∫fminfmaxABCfdf,

where *S*_SOI_(*f*) is the amplitude spectrum of the backscatter SOI, *S*_*r*_(*f*) is the reference spectrum of the backscatter signal reflected by a standard steel plate, and *f*_max_ and *f*_min_ correspond to the −6 dB effective frequency band. The central frequency is used in the calculation of the ABC.

## 3. Results

### 3.1. Backscatter Properties for Ultrasonic Propagation Perpendicular to MTD

**Figure 5:**
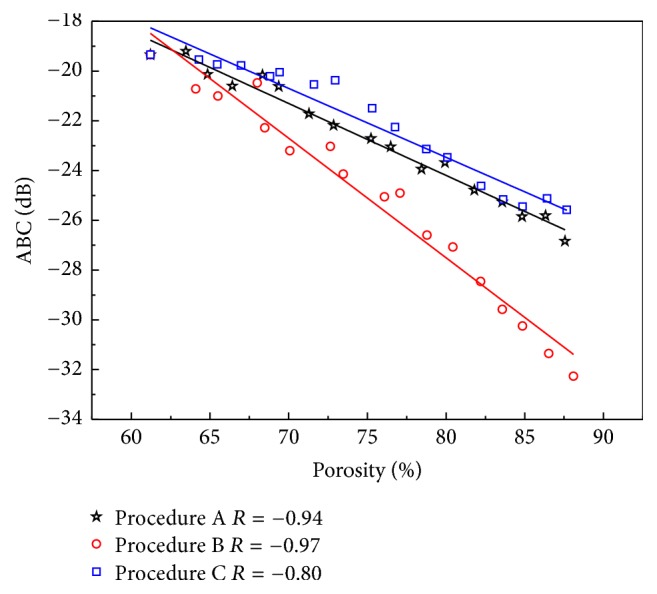
Relationships between ABC and cancellous bone porosity induced by different erosions for ultrasonic propagating perpendicular to the MTD.

Figure 5 shows the ABC versus the porosity induced by three different erosion procedures for ultrasonic propagation perpendicular to MTD. The ABC shows significant negative correlations with the porosity of the cancellous bone in all procedures (procedure A: *R* = −0.94; procedure B: *R* = −0.97; procedure C: *R* = −0.80). In the three different erosion procedures, the cancellous bone microstructure undergoes different changes, and the ultrasonic backscatter signals are different. The ABC of procedure B are the smaller than those of the other two procedures, and in procedure B the trabecular microstructural variations are parallel to MTD. The linear fittings for ABC versus the porosity of the three erosion procedures are listed in Table 2. Significant differences are observed between the slopes for the three erosion procedures (procedure A: −1.22 dB; procedure B: −0.98 dB; procedure C: −0.46 dB). The absolute values of the slope for procedure C are the smallest.

**Figure 6:**
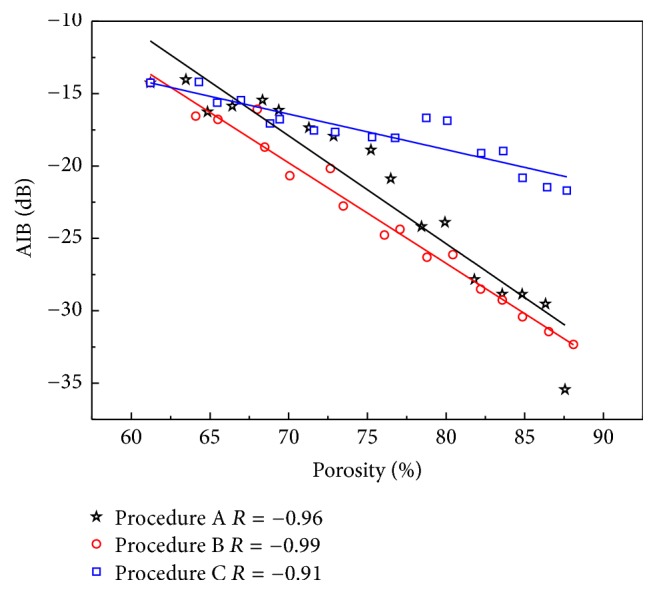
Relationships between AIB and cancellous bone porosity induced by different erosions for ultrasonic propagating perpendicular to the MTD.

The AIB is an important parameter of ultrasonic backscattering. The AIB versus the porosity of the cancellous bone induced by the different erosion procedures perpendicular to MTD is shown in Figure 6. Significant negative correlations with the porosity of the cancellous bone are observed in all procedures (procedure A: *R* = −0.96; procedure B: *R* = −0.99; procedure C: *R* = −0.91). The liner regressions between the AIB and porosity showed significant differences in the slopes for the three erosion procedures (procedure A: −0.74 dB; procedure B: −0.69 dB; procedure C: −0.25 dB).

### 3.2. Backscatter Properties for Ultrasonic Propagation along MTD

**Figure 7:**
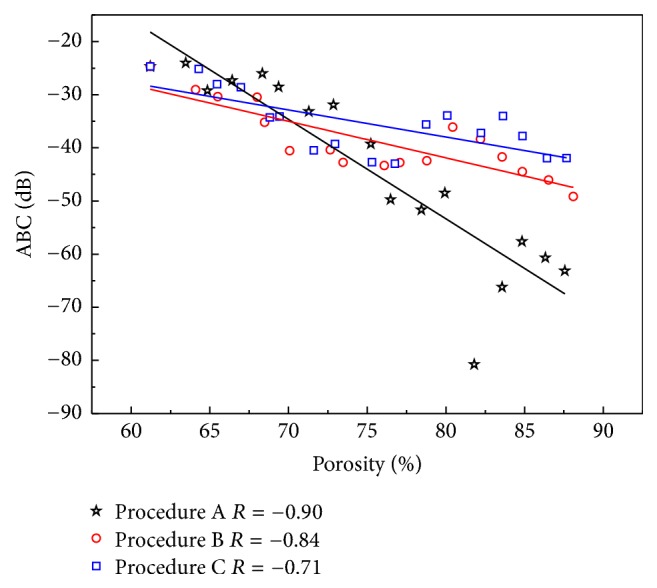
Relationships between ABC and cancellous bone porosity induced by different erosions for ultrasonic propagation along the MTD.

**Figure 8:**
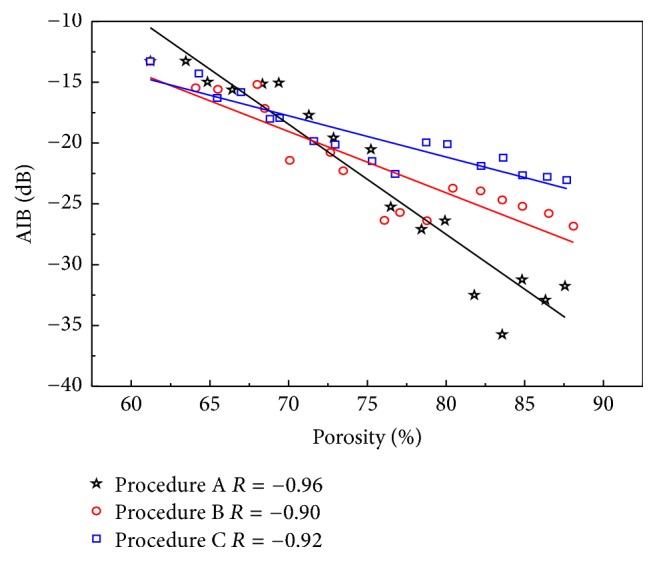
Relationships between AIB and cancellous bone porosity induced by different erosions for ultrasonic propagation along the MTD.

The ABC and AIB results versus the porosity of the cancellous bone induced by the different erosion procedures along the MTD are shown in Figures 7 and 8, respectively. Both the ABC and AIB show significant negative correlations with the porosity of the cancellous bone in all procedures (ABC: *R* = −0.90, −0.84, −0.71; AIB: *R* = −0.96, −0.90, −0.92). For the three different erosion procedures, with the increase in the cancellous bone porosity, the cancellous bone microstructure undergoes various changes. The changes in ABC and AIB are different for the same porosity. Based on the regression results listed in Table 2, the slopes for the three erosion procedures are different. Compared with the results perpendicular to the MTD, the values of ABC and AIB are smaller, and the corresponding slopes are different.

## 4. Discussion

ABC and AIB reflect the frequency-related intensity of the backscatter signal. The signal strength is mainly affected by the scattering cross section and attenuation. The reflected signal energy increases with the scattering cross section. When the ultrasonic incident direction is along the MTD, the scattering cross section is smaller than that for ultrasonic perpendicular to MTD and the values of ABC are smaller. Besides, the attenuation increases with trabecular bone length in the transmission direction. Thus, the ABC and AIB not only are influenced by bone mass or porosity but also will be influenced by trabecular microstructure of cancellous bone.

The simulations show that ABC and AIB vary differently for the three procedures with the increasing of porosity. With the notations ABC_*φ*_, AIB_*φ*_ is used to represent ABC, AIB under procedure *φ*  (*φ* = A, B, C), the simulation results show that when the ultrasonic propagation is perpendicular to MTD, both ABC and AIB show significant negative correlations with the porosity of the cancellous bone in all procedures. The slopes of the liner fitting results for the three erosion procedures (ABC: −1.22 dB, −0.98 dB, and −0.46 dB; AIB: −0.74 dB, −0.69 dB, and −0.25 dB) are different. To the same porosity, the ABC_B_ and AIB_B_ are smaller than those of the other two procedures. In procedure B, the trabecular microstructure deterioration along the MTD, the length of the trabeculae in the propagation direction, is larger than those of the other procedures, which simulate the spaceflight-induced bone loss. The results indicate that the ABC and AIB may be smallest in the case of the trabecular microstructure deterioration along the MTD when propagation is perpendicular to MTD and with the increasing porosity. The slopes of procedure C are the smallest, which simulates the age-related bone loss. Besides, the difference values of the three procedures also increase for both ABC and AIB.

The ABC_*φ*_ and AIB_*φ*_ for the three erosion procedures are also different when the propagation is along MTD, but compared to the results of propagation perpendicular to MTD, the values of ABC and AIB are smaller, the corresponding slopes of procedures A, B, and C (perpendicular MTD: ABC: −1.22 dB, −0.98 dB, −0.46 dB; AIB: −0.74 dB, −0.69 dB, −0.25 dB; along MTD: ABC: −1.87 dB, −0.69 dB, −0.51 dB; AIB: −0.9 dB, −0.5 dB, −0.34 dB) are also different, because there is a major trabecular direction in cancellous bone. The results along MTD further indicate the variability in ABC and AIB induced by the trabecular microstructure in cancellous bone and illustrate that ABC and AIB are sensitive to the trabecular microstructure.

It is the same as the experimental results in the previous study [40]; ABC and AIB both had negative correlations with the porosity. The ABC and AIB are influenced by the trabecular microstructure deterioration direction; the slops of linear fitting between them (ABC and AIB) and porosity indicate that, for the linear evaluation of BV/TV or porosity using ABC or AIB to the age-related and spaceflight-induced bone loss, the deterioration direction maybe should be considered.

In a previous study it was suggested that the ultrasound backscatter was affected by the anisotropic microstructure [19]. And to the same reason induced bone loss, to the trabecular microstructures are various, but the deterioration direction is the same. In the present study, the effect of deterioration direction is investigated. And it is significant that when the deterioration direction is considered, the liner assessment of BV/TV by the ABC or AIB is more accurate.

This study investigated the relationship between ultrasonic backscatter and cancellous bone microstructure deterioration and indicated that the ultrasonic backscatter was affected by cancellous bone microstructure deterioration direction, and we just discussed the parameters of ABC and AIB. Therefore, the study of the trabecular microstructure effect on the different ultrasonic backscatter parameters without the porosity should be elaborated upon in the future.

## 5. Conclusion

The variabilities of ABC and AIB induced by different direction deteriorations of trabecular microstructure were investigated. ABC and AIB showed significantly negative correlations with the porosity of the cancellous bone. ABC and AIB were sensitive to the trabecular microstructure; they were confined to erosion procedures from which three different direction cancellous bone microstructure changes were revealed. The ABC and AIB are affected by the trabecular microstructure deterioration direction. When using ABC and AIB accurately in the evaluation of cancellous bone mass on different reason related bone loss, the effect of the trabecular microstructure deterioration direction maybe should be considered.

## Figures and Tables

**Table 1:** Physical parameter values of cancellous bone [31].

	Trabeculae	Bone Marrow
First Lamé coefficient (GPa)	14.8	2.2
Second Lamé coefficient (GPa)	8.3	0
Density (kg/m^3^)	1960	1000
Normal resistance coefficient (s^−1^)	8 × 10^4^	75
Shear resistance coefficient (s^−1^)	8 × 10^5^	0

**Table 2:** Linear fitting for the ultrasonic backscatter parameters (ABC and AIB) versus the porosity of the three erosion procedures perpendicular and parallel to MTD^1^.

	Perpendicular to MTD			Parallel to MTD		
	Procedure A	Procedure B	Procedure C	Procedure A	Procedure B	Procedure C
ABC						
Intercept (dB)	51.59	31.16	0.02	96.27	13.10	285
Slope (dB)	−1.22	−0.98	−0.46	−1.87	−0.69	−0.51
AIB						
Intercept (dB)	34.23	28.85	0.78	44.82	16.21	5.94
Slope (dB)	−0.74	−0.69	−0.25	−0.90	−0.50	−0.34

^1^All of the *p* values are below 0.01.
